# Genome-Wide Association Study in a Lebanese Cohort Confirms *PHACTR1* as a Major Determinant of Coronary Artery Stenosis

**DOI:** 10.1371/journal.pone.0038663

**Published:** 2012-06-20

**Authors:** Jörg Hager, Yoichiro Kamatani, Jean-Baptiste Cazier, Sonia Youhanna, Michella Ghassibe-Sabbagh, Daniel E. Platt, Antoine B. Abchee, Jihane Romanos, Georges Khazen, Raed Othman, Danielle A. Badro, Marc Haber, Angelique K. Salloum, Bouchra Douaihy, Nabil Shasha, Samer Kabbani, Hana Sbeite, Elie Chammas, Hamid el Bayeh, Francis Rousseau, Diana Zelenika, Ivo Gut, Mark Lathrop, Martin Farrall, Dominique Gauguier, Pierre A. Zalloua, the FGENTCARD Consortium

**Affiliations:** 1 CEA-Genomics Institute, Centre National de Génotypage, Evry, France; 2 Centre d’Etude du Polymorphisme Humain, Paris, France; 3 The Wellcome Trust Centre for Human Genetics, University of Oxford, Headington, Oxford, United Kingdom; 4 Lebanese American University, School of Medicine, Beirut, Lebanon; 5 Bioinformatics and Pattern Discovery, IBM T. J. Watson Research Centre, New York, New York, United States of America; 6 Department of Internal Medicine, American University of Beirut, Beirut, Lebanon; 7 Division of Cardiology, Rafic Hariri University Hospital, Beirut, Lebanon; 8 IntegraGen SA, Genopole Campus 1 - Genavenir 8, Evry, France; 9 INSERM, UMRS872, Centre de Recherche des Cordeliers, Paris, France; 10 Harvard School of Public Health, Boston, Massachusetts, United States of America; University of Cincinnati, United States of America

## Abstract

The manifestation of coronary artery disease (CAD) follows a well-choreographed series of events that includes damage of arterial endothelial cells and deposition of lipids in the sub-endothelial layers. Genome-wide association studies (GWAS) of multiple populations with distinctive genetic and lifestyle backgrounds are a crucial step in understanding global CAD pathophysiology. In this study, we report a GWAS on the genetic basis of arterial stenosis as measured by cardiac catheterization in a Lebanese population. The locus of the phosphatase and actin regulator 1 gene (*PHACTR1*) showed association with coronary stenosis in a discovery experiment with genome wide data in 1,949 individuals (rs9349379, OR = 1.37, p = 1.57×10^−5^). The association was replicated in an additional 2,547 individuals (OR = 1.31, p = 8.85×10^−6^), leading to genome-wide significant association in a combined analysis (OR = 1.34, p = 8.02×10^−10^). Results from this GWAS support a central role of *PHACTR1* in CAD susceptibility irrespective of lifestyle and ethnic divergences. This association provides a plausible component for understanding molecular mechanisms involved in the formation of stenosis in cardiac vessels and a potential drug target against CAD.

## Introduction

Heart disease is a leading cause of illness, disability and death in industrialized countries, particularly in older people [1], [2]. The manifestation of CAD follows a well-choreographed series of events that includes the damaging of endothelial cells of the arteries and the gradual deposition of lipids in the sub-endothelial layers [3], [4]. CAD is a multifactorial disease, with both acquired and inherited components implicated in its etiology [4]. While most CAD risk factors can be ameliorated through lifestyle changes, such as diet and exercise, inherited causes, i.e. genetic make-up and family history of the disease, are not modifiable [5]–[7]. A small proportion of CAD cases can be attributed to rare, highly penetrant monogenic effects [8], [9], whereas most cases result from the cumulative effect of multiple susceptibility alleles and environmental factors [5], [10]–[13]. Indeed, recent GWAS have identified common variants in several novel candidate genes for cardiovascular disease, such as the *CDKN2A/2B/ANRIL* gene cluster on 9p21.3, and *PHACTR1* on chromosome 6p24.1 [14]–[16]. However, differences in lifestyle can have major impact on CAD risk and how these may influence the genetic association is largely unknown.

**Table 1:** Association of CAD stenosis categories with conventional risk factors.

		GWA phase			*p* value^1)^	Replication phase			*p* value^1)^		Total			*p* value^1)^
Stenosis level		no	mild	severe		no	mild	severe		*p* value^2)^	no	mild	severe	
Total sample size		n = 426	n = 216	n = 1307		n = 458	n = 414	n = 1675			n = 1076	n = 630	n = 2982	
Gender (%)					<0.001				<0.001	<0.001				<0.001
	Female	184 (43.2)	76 (35.2)	273 (20.9)		232 (50.7)	173 (41.8)	444 (26.5)			416 (38.7)	249 (39.5)	717 (24.0)	
	Male	242 (56.8)	140 (64.8)	1034 (79.1)		226 (49.3)	240 (58.0)	1230 (73.4)			468 (43.5)	380 (60.3)	2264 (75.9)	
Age (SD)		56.3 (11.7)	62.6 (11.4)	63.6 (10.8)	<0.001	56.1 (10.9 )	60.2 (11.1 )	63.2 (10.7 )	<0.001	0.11	56.2 (11.3)	61.0 (11.3)	63.4 (10.7)	<0.001
History of type 2 Diabetes (%)		70 (16.4)	45 (20.8)	458 (35.0)	<0.001	84 (18.3)	101 (24.4)	610 (36.4)	<0.001	0.2	154 (14.3)	146 (23.2)	1068 (35.8)	<0.001
History of Hypertension (%)		183 (43.0)	117 (54.2)	763 (58.4)	<0.001	250 (54.6)	261 (63.0)	1129 (67.4)	<0.001	<0.001	433 (40.2)	378 (60.0)	1892 (63.4)	<0.001
History of Hyperlipidemia (%)		155 (36.4)	94 (43.5)	685 (52.4)	<0.001	178 (38.9)	190 (45.9)	882 (52.7)	<0.001	0.52	333 (30.9)	284 (45.1)	1567 (52.5)	<0.001
Family history of CAD (%)		273 (64.1)	130 (60.2)	928 (71.0)	<0.001	187 (40.8)	166 (40.1)	711 (42.4)	0.626	<0.001	460 (42.8)	296 (47.0)	1639 (55.0)	<0.001

Analysis was done in GWA (discovery) and replication phases separately and combined (total). No, mild, and severe correspond to CAD severity categories 1, 2 and 3. **^1)^** *p* value indicating association of risk factors with CAD categories. **^2)^** *p* value indicating association of risk factors with different phases. See Methods section for details.

Replication of the GWAS results for the most significantly associated polymorphisms in multiple populations with distinctive genetic and lifestyle backgrounds may provide deeper understanding of the pathophysiology of a multifactorial disease like CAD. The current trend in GWA studies relies on genetic analyses of ever increasingly large numbers of individuals, with meta-analysis of cohorts that often are from different phenotypic, genetic and environmental backgrounds as well as with diverse ascertainment schemes [17]–[19]. A complementary approach lies in the use of smaller but phenotypically well-characterized populations to probe genetic determinants of CAD intermediary phenotypes. Such populations can be especially informative when they have a specific genetic and environment backgrounds. We have carried out investigations of a genome-wide set of genetic variations associated with severity of CAD measured by the degree of coronary stenosis in the Lebanese population that displays limited admixture levels, specific genetic background, and a generally Mediterranean diet and lifestyle. A study has shown that twenty percent of the general Lebanese population has high total cholesterol and LDL levels and that the prevalence of overweight individuals in this population aged 20 years and above is 57.7% in men and 49.4% in women despite the healthy Mediterranean diet consumed [20]. Further, it implicated several risk factors including age, gender, hypertension, fasting blood glucose, lipoprotein (a), and a positive family history of CAD as important predictors of CAD in the Lebanese population. Since the majority of these factors possess genetic determinants, it is of interest to conduct genome-wide association studies and evaluate the association of gene polymorphisms with CAD in this population and assess their contribution to the disease development and prevention.

We carried out a discovery GWAS in 1,949 individuals with coronary angiogram data followed by an independent replication experiment in 2,547 subjects. We confirmed association with common variants in *PHACTR1* that were significantly associated with this disease.

## Materials and Methods

### 1. Study Subjects

The study subjects consisted of 4,741 individuals who underwent cardiac catheterization following a single consistent and stringent recruitment protocol between August, 2007 and June, 2009 at several hospitals in Lebanon [21]. Catheterization was prompted for myocardial infarction (MI) (13.1%) as diagnosed by electrocardiogram and high troponin levels, unstable angina (30.3%), or other reasons, such as stable angina, or heart failure, or reversible ischemia by stress testing (56.5%). All patients underwent coronary catheterization by Judkins’ technique. The four main coronary arteries: the left main artery (LMCA), the left anterior descending artery (LAD), the left circumflex artery (LCx), and the right coronary artery (RCA) were visualized from different angles by angiography. Two experienced interventional cardiologists reviewed the coronary angiograms independently. The stenotic lesions in these vessels were assessed and recorded as a percentage of coronary blockage. The extent of the coronary lesion was estimated visually by comparing the reduction in the diameter of the narrowed vessel to a proximal assumed normal arterial segment. Cardiologists performing the coronary angiography collected a 20 mL blood sample from the arterial access site of patients who provided a written consent for the whole study that included blood collection and genetic analysis. Trained healthcare professionals collected further data on the socio-demographic background of all patients. Annotations were coded from medical charts for additional data such as laboratory tests, prescribed medications, and presence of other diseases and conditions. Genomic DNA was extracted using a standard phenol extraction procedure. The Institutional Review Board (IRB) at the Lebanese American University approved the study protocol.

**Figure 1:**
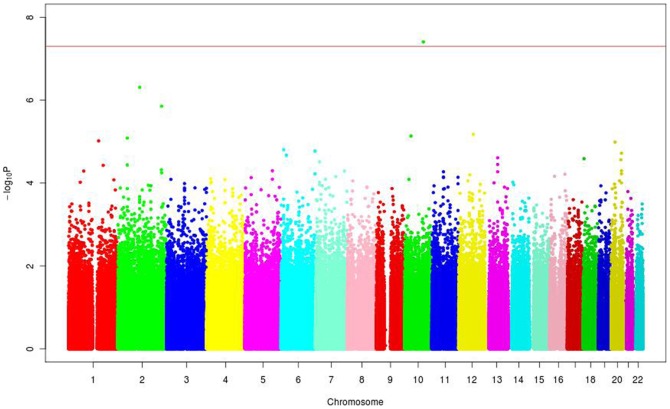
Manhattan plot showing results of a GWA analysis in 1949 Lebanese patients for 513,079 SNPs. The Y-axis corresponds to the significance of the association (-log_10_ p-values). The X-axis represents the physical location of the variant colored by chromosome.

**Figure 2:**
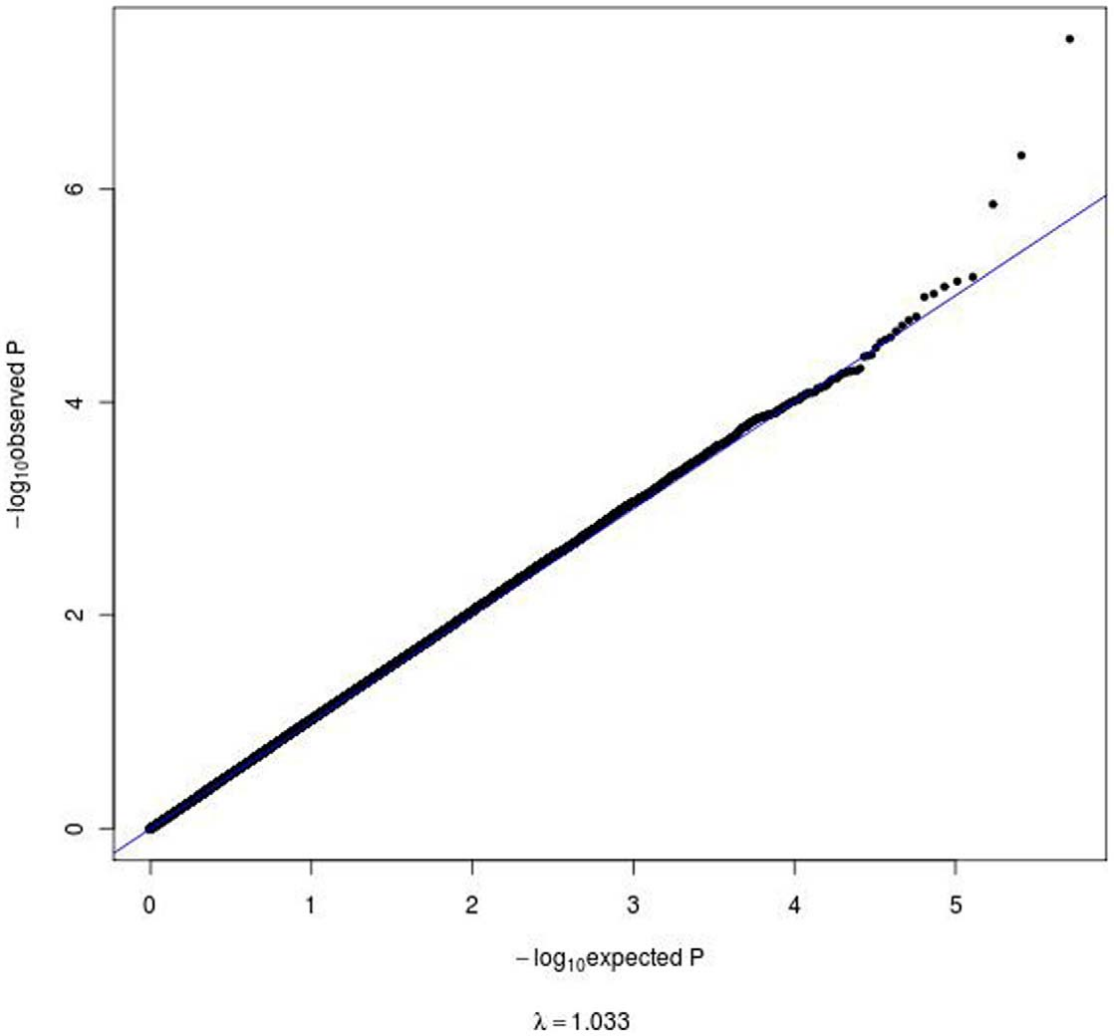
Quantile-Quantile plot of the GWAS results. In this plot, each dot corresponds to a SNP tested for association where the observed –log_10_ p values, shown by vertical axis, were plotted by the expected –log_10_ p values under the null hypothesis. Upper right dots with higher observed significance than expected represent candidate variants for association with the phenotype tested (CAD category 1, control subjects; CAD category 2, patients with ≤50% stenosis in any coronary artery; CAD category 3, patients with >50% stenosis in any of the coronary arteries). The genomic control ratio (λ) was 1.033, indicating the lack of strong effect of systematic error such as population stratification.

For the primary analysis, patients with a normal angiogram with no visible lesions in any of the four coronary arteries were classified as CAD category 1 and considered as control subjects. Patients with coronary artery stenosis were classified into two categories: CAD category 2 comprised patients with ≤50% stenosis (moderate) in any coronary artery, and CAD category 3 comprised patients with >50% stenosis (severe) in any of the coronary arteries [22]. For the genome-wide association analysis with CAD degree of stenosis, the comparisons were done among subjects in each of the three CAD categories. For the genome-wide association analysis with site of stenosis for each of the 4 coronary arteries, patients in CAD categories 1and 2 were compared to CAD category 3 patients.

### 2. Genotyping

#### 2.1 Whole genome

For the initial discovery phase of the study, DNA samples of the first 2,002 recruited individuals were utilized for whole genome genotyping. A total of 1,210 and 792 individuals were genotyped by Illumina Human610-Quad BeadChip and Illumina Human660W-Quad BeadChip respectively (552 510 overlapping SNPs), as part of the Functional Genomic Diagnostic Tools for Coronary Artery Disease project initiative (FGENTCARD) [21]. Out of the 2,002 genomic DNA samples genotyped, 1,949 non-duplicated samples with quality filtered genotyping data were retained.

#### 2.2 Targeted genotyping

For replication analyses, we have selected (1) all SNPs with p-value <10^−5^ (n = 7) from primary analysis (GWAS phase), (2) SNPs in previously identified candidate genes that showed a p-value <10^−3^ (n = 8) in the GWAS phase and (3) SNPs located in a region of 500 Kb around the eight candidate genes (n = 5). These SNPs were re-genotyped by mass spectrometry (Sequenom, CA) in the initial GWAS sample. In addition, 2,739 independent replication samples from the same ascertainment centers were genotyped by Sequenom mass spectrometry. From the 2,739 genotyped samples, 192 individuals were excluded due to missing clinical and demographic information and the resulting 2,547 samples were analyzed. In addition to the above 20 SNPs, rs12526453 in the PHACTR1 region that has been previously identified to be associated with early-onset MI, and not present on the Illumina chip for the GWAS phase, was selected to be genotyped in the replication cohort.

**Table 2:** Cumulative logit analyses for the most significant SNPs in the GWA phase and the corresponding scores in the replication phase as well as the combined effect from the meta-analysis sorted by the level of significance in the meta-analysis.

									GWA phase								Replication phase						Meta-analysis		
									Frequency of effect allele								Frequency of effect allele								
SNP	Candidate gene	Chr	bp position	Effect allele		Other allele		n	no	mild	severe		OR	95%CI	*p*	n	no	mild	severe	OR	95%CI	*p*	OR	95%CI	*p-*meta
rs9349379	PHACTR1	6	13011943	G		A		1940	0.342	0.373	0.405		1.374	1.19–1.59	1.57E-05	2529	0.349	0.35	0.408	1.313	1.16–1.48	8.85E-06	1.338	1.22–1.47	8.02E-10
rs2327620	PHACTR1	6	13015577	A		G		1947	0.52	0.5	0.47		0.788	0.69–0.91	7.37E-04	2514	0.492	0.515	0.457	0.819	0.73–0.92	8.07E-04	0.806	0.74–0.88	2.39E-06
rs11186734		10	93647236	T		C		1948	0.116	0.093	0.065		0.508	0.4–0.65	3.94E-08	2516	0.064	0.074	0.066	0.992	0.79–1.25	9.45E-01	0.715	0.61–0.85	8.53E-05
rs4674220	TNS1	2	218450247	G		A		1945	0.3	0.365	0.38		1.351	1.16–1.57	5.66E-05	2531	0.317	0.324	0.34	1.103	0.97–1.25	1.26E-01	1.2	1.09–1.32	2.00E-04
rs206184	MACC1-ITGB8	7	20323910	C		T		1945	0.326	0.286	0.386		1.332	1.15–1.54	1.10E-04	2529	0.361	0.375	0.383	1.099	0.97–1.24	1.27E-01	1.188	1.08–1.3	3.07E-04
rs13006511	GCC2-LIMS1	2	108405522	C		T		1944	0.173	0.149	0.107		0.606	0.5–0.73	4.88E-07	2525	0.125	0.137	0.134	0.985	0.83–1.17	8.61E-01	0.796	0.7–0.9	4.35E-04
rs890049	TNS1	2	218453131	T		C		1948	0.305	0.377	0.397		1.428	1.23–1.65	1.40E-06	2497	0.35	0.37	0.364	1.027	0.91–1.16	6.66E-01	1.174	1.07–1.29	7.96E-04
rs6974002	MACC1-ITGB8	7	20333053	G		A		1948	0.235	0.227	0.301		1.402	1.19–1.65	3.09E-05	2490	0.275	0.309	0.294	1.063	0.93–1.21	3.51E-01	1.184	1.07–1.31	9.68E-04
rs7964864	KCNC2	12	72976559	C		A		1945	0.075	0.09	0.127		1.723	1.35–2.2	6.71E-06	2517	0.111	0.108	0.125	1.076	0.89–1.30	4.44E-01	1.28	1.1–1.49	1.14E-03
rs2577625	GCC2-LIMS1	2	108567088	T		C		1936	0.501	0.447	0.43		0.779	0.68–0.89	2.94E-04	2499	0.451	0.464	0.455	0.962	0.86–1.08	5.20E-01	0.878	0.8–0.96	4.19E-03
rs6455455	SMOC2-THBS2	6	169076823	C		T		1946	0.147	0.137	0.094		0.631	0.51–0.78	1.70E-05	2525	0.116	0.11	0.118	1.034	0.86–1.24	7.28E-01	0.83	0.72–0.95	8.48E-03
rs4708388	SMOC2-THBS2	6	169035590	A		G		1947	0.146	0.134	0.095		0.656	0.54–0.8	6.02E-05	2531	0.113	0.116	0.116	1.026	0.86–1.23	7.85E-01	0.842	0.74–0.96	1.29E-02
rs16891359	Histone cluster	6	26256979	C		T		1945	0.018	0.042	0.053		2.259	1.51–3.38	2.16E-05	2518	0.047	0.062	0.054	1.018	0.79–1.32	8.90E-01	1.288	1.04–1.6	2.31E-02
rs7919192		10	93650707	C		T		1944	0.415	0.337	0.347		0.789	0.69–0.91	9.40E-04	2505	0.344	0.371	0.351	1.001	0.89–1.13	9.89E-01	0.903	0.82–0.99	2.91E-02
rs1543121	NTM	11	130903744	T		G		1942	0.091	0.095	0.144		1.553	1.24–1.94	7.28E-05	2512	0.111	0.14	0.113	0.926	0.77–1.11	4.04E-01	1.137	0.99–1.31	7.46E-02
rs4936137	NTM	11	130901361	A		G		1947	0.093	0.097	0.144		1.534	1.23–1.92	1.04E-04	2516	0.111	0.142	0.113	0.917	0.76–1.10	3.45E-01	1.125	0.98–1.29	1.02E-01
rs7294453	KCNC2	12	72938064	G		T		1942	0.056	0.067	0.094		1.579	1.2–2.08	8.39E-04	2517	0.087	0.082	0.086	0.923	0.75–1.14	4.63E-01	1.126	0.95–1.33	1.67E-01
rs9805437	FGF14	13	101634297	G		A		1946	0.108	0.088	0.143		1.409	1.14–1.75	1.41E-03	2518	0.143	0.151	0.13	0.887	0.75–1.05	1.60E-01	1.057	0.93–1.21	4.16E-01
rs4535467	CDH6	5	31245124	A		G		1947	0.081	0.076	0.115		1.618	1.27–2.07	7.41E-05	2517	0.127	0.126	0.109	0.842	0.70–1.01	6.38E-02	1.061	0.92–1.23	4.28E-01
rs7995765	FGF14	13	101627418	T		C		1946	0.092	0.065	0.131		1.522	1.21–1.92	2.85E-04	2532	0.131	0.13	0.113	0.843	0.71–1.00	5.70E-02	1.044	0.91–1.2	5.49E-01

Alleles follow forward strand of NCBI36 reference genome. OR (odds ratio) was calculated from major homozygote as a base line. 95%CI is a 95% confidential interval of OR.

**Figure 3:**
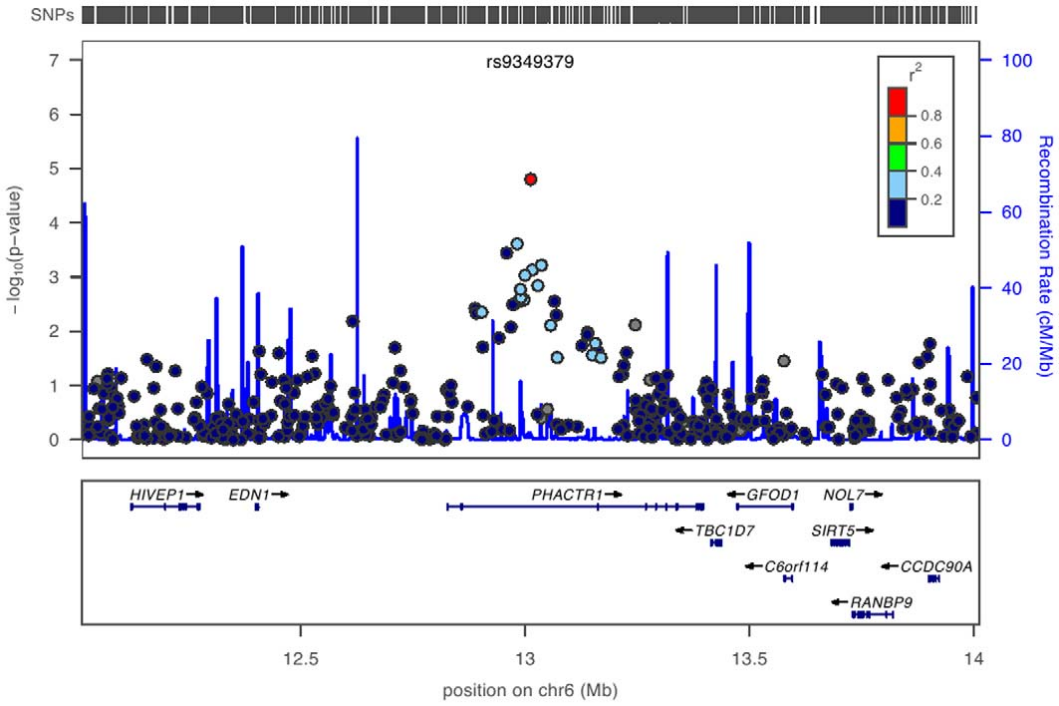
Map around the PHACTR1 locus on chromosome 6 showing strong evidence of association with coronary artery stenosis. The upper vertical bars correspond to the location of all markers tested from the SNP array chip in the area. The X-axis indicates the chromosomal position in base-pairs on chromosome 6. Recombination rate is presented as a continuous blue line, while individual markers are represented by a circle filled with a color corresponding to the extent of LD with the key marker rs9349379 (in red) from dark blue (r^2^ = 0) to red (r^2^ = 1). Grey filled circles refer to SNPs with no LD information. The lower part represents the location of the genes with corresponding exons and the direction of transcription indicated by arrows. The figure was generated with LocusZoom using CEU from HapMap release 22 as reference.

**Table 3:** Results of the cumulative logit analyses for the most significant SNPs in the combined exploratory and replication phases sorted by the level of significance using specific vessel stenosis as outcome variables.

					LMCA(n = 403)			LAD(n = 366)			LCx(n = 64)		RCA(n = 473)		
SNP	Candidate gene	Effect allele	Other allele	OR	95%CI	*p*	OR	95%CI	*p*	OR	95%CI	*p*	OR	95%CI	*p*
rs9349379	PHACTR1	G	A	1.14	1.01–1.28	2.75E-02	1.327	1.22–1.44	6.04E-12	1.187	1.10–1.29	2.89E-05	1.23	1.14–1.33	4.38E-07
rs2327620	PHACTR1	A	G	0.903	0.81–1.01	8.29E-02	0.803	0.74–0.87	4.98E-08	0.864	0.80–0.93	2.39E-04	0.864	0.80–0.93	2.48E-04
rs206184	MACC1-ITGB8	C	T	1.082	0.96–1.22	1.86E-01	1.152	1.06–1.25	5.87E-04	1.125	1.04–1.22	4.12E-03	1.085	1.00–1.18	4.59E-02
rs4674220	TNS1	G	A	1.093	0.97–1.23	1.42E-01	1.103	1.02–1.20	2.07E-02	1.166	1.07–1.27	2.77E-04	1.133	1.04–1.23	3.16E-03
rs11186734		T	C	0.769	0.60–0.98	3.26E-02	0.764	0.66–0.89	4.46E-04	0.883	0.76–1.03	1.07E-01	0.767	0.66–0.89	6.65E-04
rs7964864	KCNC2	C	A	1.129	0.95–1.34	1.70E-01	1.077	0.95–1.22	2.46E-01	1.163	1.03–1.32	1.73E-02	1.114	0.98–1.26	8.92E-02
rs890049	TNS1	T	C	1.036	0.92–1.17	5.56E-01	1.126	1.04–1.22	4.28E-03	1.128	1.04–1.22	3.47E-03	1.113	1.03–1.21	9.24E-03
rs6974002	MACC1-ITGB8	G	A	0.994	0.88–1.13	9.23E-01	1.173	1.08–1.28	3.29E-04	1.071	0.98–1.17	1.20E-01	1.04	0.95–1.13	3.71E-01
rs13006511	GCC2-LIMS1	C	T	0.939	0.79–1.11	4.66E-01	0.898	0.8–1.00	6.08E-02	0.957	0.85–1.07	4.52E-01	0.928	0.83–1.04	1.96E-01
rs4708388	SMOC2-THBS2	A	G	0.787	0.65–0.95	1.39E-02	0.874	0.77–0.99	2.92E-02	0.833	0.74–0.94	3.51E-03	0.859	0.76–0.97	1.44E-02
rs6455455	SMOC2-THBS2	C	T	0.899	0.75–1.08	2.64E-01	0.866	0.77–0.98	2.26E-02	0.841	0.74–0.95	6.89E-03	0.848	0.75–0.96	9.40E-03
rs2577625	GCC2-LIMS1	T	C	1.06	0.95–1.19	3.10E-01	0.944	0.87–1.02	1.48E-01	0.934	0.86–1.01	8.76E-02	0.898	0.83–0.97	6.87E-03
rs16891359	Histone cluster	C	T	1.099	0.85–1.41	4.63E-01	1.094	0.91–1.31	3.29E-01	1.159	0.97–1.39	1.08E-01	1.162	0.97–1.39	9.58E-02
rs7919192		C	T	0.976	0.87–1.10	6.85E-01	0.919	0.85–1.00	4.12E-02	0.944	0.87–1.02	1.64E-01	0.917	0.85–0.99	3.49E-02
rs1543121	NTM	T	G	1.196	1.02–1.41	3.22E-02	1.032	0.92–1.16	6.04E-01	1.053	0.93–1.19	3.94E-01	1.129	1.00–1.27	4.39E-02
rs7294453	KCNC2	G	T	1.093	0.90–1.33	3.82E-01	1.048	0.91–1.21	5.26E-01	1.065	0.92–1.23	3.84E-01	1.014	0.88–1.17	8.45E-01
rs4936137	NTM	A	G	1.16	0.98–1.37	7.90E-02	1.023	0.91–1.15	7.03E-01	1.056	0.94–1.19	3.67E-01	1.126	1.00–1.27	4.95E-02
rs9805437	FGF14	G	A	1.145	0.97–1.35	1.00E-01	1.034	0.92–1.16	5.69E-01	1.107	0.99–1.24	8.30E-02	1.077	0.96–1.21	1.98E-01
rs4535467	CDH6	A	G	0.963	0.80–1.16	6.86E-01	1.208	1.06–1.37	3.61E-03	1	0.88–1.13	9.96E-01	0.997	0.88–1.13	9.60E-01
rs7995765	FGF14	T	C	1.105	0.93–1.31	2.52E-01	1.059	0.94–1.20	3.53E-01	1.2	1.06–1.35	2.98E-03	1.072	0.95–1.21	2.59E-01

The number of patients with site specific stenosis is marked next to the vessel type. Patients in each stenosis site category were compared to the n = 642 controls available.

Alleles follow forward strand of NCBI36 reference genome. LMCA: left main artery; LAD: left anterior descending artery; LCx: left circumflex artery; RCA: right coronary artery.

### 3. Statistical Analysis

We characterized subjects using ordered (e.g. degree of stenosis) and unordered (e.g. site of stenosis) categorical measures, and continuous variables for analysis. The study was divided into an initial discovery phase based on 1,949 individuals and 2,547 individuals for further genotyping to confirm genome-wide signals. The associations of the CAD stenosis categories with conventional risk factors were examined by Fisher’s exact test for count data, and by ANOVA for continuous valued response variables. We also evaluated the association of these risk factors between GWA and replication phases. For every risk factor, we tested for differences in proportion at each degree of stenosis between the two phases. A one-degree of freedom log-linear analysis on the phase effect was used for the qualitative traits, and two-way ANOVA for the quantitative traits.

All SNPs with over 98% genotyping success rate, minor allele frequency of above 1% and that are in Hardy-Weinberg equilibrium (p>1×10^−7^) were included in the analysis. All 20 SNPs selected were in HWE (p-value >1E-4) and had a MAF more than or equal to 5%. The population substructure (ancestry analysis) was examined by Principal Component Analysis (PCA) using the EIGENSTRAT software [23].

Analyses were based on an additive genetic model. We employed the cumulative logit analysis of the proportional odds model [24] for CAD category data, which was expressed as ordinal categorical data: no, mild and severe stenosis. Three covariates, indicator of genotyping BeadChip, sex, and age at investigation of coronary angiogram, were included in the model as a primary analysis. P values were calculated by likelihood ratio test. A p-value <5.0×10^−8^ was considered to be genome-wide significant. The replication set included the same categorical phenotype allowing analysis with the same cumulative logit model. The results of the discovery and replication phases were combined by fixed effect meta-analysis using “rmeta” package in R.

## Results

The study population has a mean age of 61.56 (±11.33), 69.2% of the individuals were males with a majority (73%) suffering from >50% stenosis in at least one of the 4 main coronary arteries (CAD category 3) (Table 1). CAD category 3 disease was manifested in 78% of the diabetic subgroup. Similar patterns of disease were observed in those suffering from hypertension, hyperlipidemia or positive family history of CAD. As expected, individuals with severe stenosis showed the highest frequency of the traditional risk factors compared to patients with no or mild stenosis. The results were statistically significant (Table 1).

### 1. GWAS Results

To map gene loci associated with coronary artery stenosis, SNPs genotyped data in the discovery cohort of 1,949 individuals were tested for association with stenosis category phenotypes. Of the 513,079 successfully genotyped SNPs that overlapped in the two genotyping BeadChips, only one SNP, rs11186734 on chromosome 10, showed genome-wide significance (OR = 0.51, p = 3.94×10^−8^) for association in the discovery cohort adjusted for indicator of genotyping BeadChip, sex, and age at investigation of coronary angiogram (Figure 1). The genomic control inflation factor (λ) [25], which compares observed association statistics against the expected distribution was 1.033 suggesting no systematic over-dispersion of the association statistics (Figure 2). Furthermore, the incorporation of the first 10 components derived from the PCA ancestry analysis as covariates into the association did not affect the results appreciably. Other SNPs showing trend of association were rs13006511 (OR = 0.61, p = 4.88×10^−7^) on 2q12, rs890049 (OR = 1.43, p = 1.40×10^−6^) on 2q35 and rs7964864 (OR = 1.72, p = 6.71×10^−6^). The SNP rs890049 maps to the tensin 1 (*TNS1*) locus encoding a protein involved in the remodeling of the extracellular matrix. An additional nearby SNP (rs4674220) in *TNS1* showed p-value of 5.66×10^−5^ (OR = 1.35). Interestingly, rs9349379 on 6p24, located in the gene encoding the protein phosphatase 1 and actin regulator 1 (PHACTR1), previously associated with early-onset MI[14]–[16], showed a p-value of 1.57×10^−5^ (OR = 1.37). None of the SNPs in other genomic regions previously associated with cardiovascular disease showed similarly strong association with the degree of coronary stenosis in our analysis [26].

### 2. Replication Results

In order to confirm the association, a selection of 20 SNPs were genotyped for replication with the Sequenom technology in the entire cohort (n = 4,496). Concordance for genotypes between the Illumina GWAS and the Sequenom data was >99% for all SNPs. With the notable exception of SNPs in the *PHACTR1* locus, none of the other SNPs showed any improvement of the results in the meta-analysis (Table 2). The SNP rs9349379 (Figure 3) showed strong association in the replication phase (OR = 1.31, p = 8.85×10^−6^), and its significance was improved in the meta-analysis (OR = 1.34, p = 8.02×10^−10^). Another variant in *PHACTR1*, rs12526453, which major allele C was previously reported to be associated with increased risk (OR = 1.15) of early-onset MI [15], [16], was not in the exploratory genome-wide BeadChip, but part of our replication effort (OR = 0.82, p = 8.9×10^−4^ for the minor allele G). Interestingly, our at-risk allele G of rs9349379 (freq = 0.39 in the replication cohort) occurred in the background of the common at-risk allele C of rs12526453 (freq = 0.62 in the replication cohort) showing strong Linkage Disequilibrium in our replication cohort at the haplotype level only (D’ = 0.98, r^2^ = 0.37). Testing for independence between these 2 signals with a multilocus analysis, we validated that our new variant rs9349379 explained most of the association observed in rs12526453; the size of its effect dropping from OR = 1.22 to OR = 1.05. Most importantly, the SNP rs9349379 still showed strong association with degree of stenosis independent of MI status (OR = 1.34, p = 2.94×10^−9^). The respective significance of SNPs in the *PHACTR1* locus was further elucidated in the context of regional recombination and LD (Figure 3) [27].

The SNPs were also tested for stenosis association with each of the four main coronary arteries. Sorted by arteries, rs9349379 in *PHACTR1* most strongly impacted LAD, with an odds ratio of 1.33 (95%CI = 1.22–1.44, p = 6.04×10^−12^), followed by RCA and LCx (Table 3).

## Discussion

Here we report the first genome-wide scan for genetic susceptibility to variable degrees of coronary artery stenosis in a Middle East population. We demonstrate strong association with variants in the *PHACTR1* gene that was previously implicated in early-onset MI [14]–[16]. GWA studies conducted in ethnically distinct and geographically distant populations with varying dietary habits, environmental exposures and differing cultures provide useful comparisons yielding knowledge about the contribution of specific genetic variants to increasingly prevalent diseases worldwide [16], [28], [29]. The design methodologies followed in these studies often display distinctive inclusion criteria and a variety of parameters have to be taken into account to carry out the necessary comparative analysis and interpretation. Variability in study enrollment, such as family-based, cohort, and case-control comparison strategies, as well as the use of different inclusion and exclusion criteria, with variations applied even between screening and validation stages of individual studies, all impact risk estimates. Most importantly, CAD outcomes are classified by varying standards. While some use coronary angiography to firmly verify the disease status, others consider healthy individuals from the general population as controls [16], [28], [29]. One of the strengths of our study lies in the phenotypic characterization that is based on stringent case-control definition criteria, whereby the percent of stenosis in all four coronary arteries was determined based on angiographic visualization. This study design excluded all asymptomatic individuals with angiographic CAD from the control group and therefore minimized misclassification issues that may arise when using unscreened control samples.

The meta-analysis of the exploratory and the replication sets showed strong and genome-wide significant evidence for association of SNP rs9349379 in *PHACTR1* with arterial stenosis in all arteries. Variants in *PHACTR1* were originally associated with early-onset MI in a study published by the MI Genetics consortium [14]–[16]. The most significant SNP in these studies, rs12526453 [15], [16], was not part of our discovery, but showed association in the replication set. However, its effect dropped after taking into account the variant rs9349379 that appears on the same background haplotype (D’ = 0.98, r^2^ = 0.37). Our results confirm recent data [14] that suggests that rs9349379 may be a better marker for the association than rs12526453. The role of PHACTR1 in the pathophysiolgy of cardiovascular disease remains to be elucidated. PHACTR1 is a regulator of protein phosphatase 1 (PP1), an enzyme that regulates endothelial nitric oxide [30], an important modulator of cardiovascular disease [31]. Furthermore, PP1 activity was shown to be elevated in patients with end-stage heart failure [32].

In accordance with two recent publications [14], [16], our results confirm that *PHACTR1* variants are associated with degree of stenosis independent of MI. Indeed, when specific major vessels were used as a stand-alone outcome variable, the association was highest with the left anterior descending artery (LAD; OR = 1.33, p = 6.04×10^−12^) and lowest with the left main artery (LMCA; OR = 1.14, p = 2.75×10^−2^) (Table 3). The association was significantly increased proportionate to number of vessels with stenosis, reaching p = 7.27×10^−10^ when stenosis was present in all 4 vessels (data not shown). Since stenosis in the coronary arteries is a major risk factor for MI, variants in *PHACTR1* may at least in part contribute to MI risk through an involvement in the formation of stenosis in the cardiac vessels independently of a role in Ca^2+^ homeostasis of the heart.

The association with rs9349379 remained significant after adjustment for gender, hypertension, dyslipidemia, and diabetes suggesting that these risk factors are not major contributors to the observed association (data not shown). This variant remains significant across different structured populations with diverse dietary and genetic profiles, which demonstrates its strong genetic component in the disease.

It is noteworthy that our GWAS did not show strong evidence for association with degree of stenosis with other previously identified gene variants for CAD. In part this may be due to sample size. The number of individuals with no stenosis was relatively modest in our study. Another factor may be that the control group was somewhat younger on a whole and it cannot be ruled out that some of these individuals may indeed develop atherosclerosis in the future. These factors may have influenced our power to detect associations. Some of the previously reported SNPs are not present and are not well tagged by other markers on the Illumina array used in this study. Also the role of the previously associated SNPs may differ among populations with different lifestyle patterns, or their distribution is such that significant associations cannot be assessed with equal power in the different populations studied.

Although they did not reach the same level of significance, many of the formerly reported variants showed nominally significant p values in the same direction as those reported [26]. The 9p21 locus that was shown to be associated with coronary heart disease in more than one GWAS [16], [28], [33], in particular variant rs4977574, did not show genome-wide significance for association with CAD (PCA adjusted p = 0.0107) or MI (p = 0.00448) in our study population [28]. Interestingly, a candidate gene approach was used to test association of candidate SNPs with CAD and/or MI in genotypic and allelic association models using logistic regression [26]. Results showed that the variant rs4977574 in *CDKN2A*-*CDKN2B* in the 9p21 locus was significantly associated with MI (OR = 1.33, p = 0.0086) in the Lebanese population. Association was detected after adjustment for confounding risk factors.

A parallel approach was conducted on a list of 20,225 variants in 88 previously published genes for association in our Lebanese cohort. The study was based on our genome-wide genotype data set, with imputation across the whole genome to CEU HapMap population as a reference. This approach identified a significant association of two new loci with CAD (*CDKAL1* and *PTPRD*), in addition to the *CXCL12* locus. Further, we identified *ST6GAL1* as having a protective effect against CAD/MI [26].

Our results contribute to ongoing efforts aiming at identifying genes associated with the multifaceted CAD and can provide important perspectives in disease diagnosis as well as new avenues for drug discovery.
